# A Wax Ball in the Vagina Thirty Years

**Published:** 1881-10

**Authors:** 


A WAX BALL IN THE VAGINA THIRTY YEARS.—A correspondent of the *American Medical Bi-Weekly* gives an interesting account of a German lady carrying a wax ball (size unknown), in her vagina for thirty years, without inconvenience. It finally produced death by causing "inflammation all around the lower portions of the vagina, including the neck of the bladder." After her last confinement (over thirty years ago), an old midwife placed it there, and said if ever removed it would cause death! The patient would not submit to the operation of taking it out.

